# Paramedic-Performed Prehospital Tele-Ultrasound: A Powerful Technology or an Impractical Endeavor? A Scoping Review

**DOI:** 10.1017/S1049023X23006234

**Published:** 2023-10

**Authors:** Rachel Shi, Javier Rosario

**Affiliations:** University of Central Florida College of Medicine, Orlando, Florida, USA

**Keywords:** emergency medicine, paramedics, prehospital, tele-ultrasound

## Abstract

Ultrasound with remote assistance (tele-ultrasound) may have potential to improve accessibility of ultrasound for prehospital patients. A review of recent literature on this topic has not been done before, and the feasibility of prehospital tele-ultrasound performed by non-physician personnel is unclear. In an effort to address this, the literature was qualitatively analyzed from January 1, 2010 – December 31, 2021 in the MEDLINE, EMBASE, and Cochrane online databases on prehospital, paramedic-acquired tele-ultrasound, and ten articles were found. There was considerable heterogeneity in the study design, technologies used, and the amount of ultrasound training for the paramedics, preventing cross-comparisons of different studies. Tele-ultrasound has potential to improve ultrasound accessibility by leveraging skills of a remote ultrasound expert, but there are still technological barriers to overcome before determinations on feasibility can be made.

## Introduction

Point-of-care ultrasound (POCUS) has found numerous applications in ambulances, helicopters, wilderness, and other resource-limited settings and provides valuable insights into a patient’s disease severity, injury pattern, and underlying health conditions, which reduces time-to-diagnosis and ultimately affects clinical decision making and patient outcomes.^1^ In recent years, the technology has become more accessible and increasingly implemented in the prehospital setting, especially in Europe where it has proven to be feasible in ground transport, air medical services, and other limited-resource environments.^2,3^ Numerous protocols that incorporate POCUS are now used to rapidly evaluate time-sensitive, life-threatening conditions (eg, Bedside Lung Ultrasound in Emergency [BLUE]/Fluid Administration Limited by Lung Sonography [FALLS] for respiratory distress;^4^ extended Focused Assessment with Sonography for Trauma [FAST]/Rapid Ultrasound for Shock and Hypotension [RUSH] exams for trauma in the thorax and abdomen^5^). In addition to high-acuity and shock conditions that may benefit more from prehospital ultrasound-supported interventions,^6^ POCUS has uses in ultrasound-guided vascular access,^7,8^ fracture detection,^9,10^ esophageal intubations,^11^ endotracheal tube placement,^12^ and measurement of optic nerve sheath diameter in cases of traumatic brain injury.^13,14^

As a highly user-dependent technology, ultrasound operation and interpretation require sufficient training and knowledge. Prior to 2010, ultrasound was typically performed by physicians in the hospital setting or helicopter Emergency Medical Service (EMS) systems.^6^ More recently, evidence has shown that an acceptable standard of competency of prehospital ultrasound can be taught relatively quickly to non-physician personnel such as paramedics.^15–18^ This is particularly important since most prehospital care teams consist of one or two non-physician personnel. However, few paramedics have had training in ultrasound. Training, skill retention, and continuing education can be laborious and involves a combination of theory, hands-on practice, and numerous clinical examinations, ideally with supervision.^19^ Barriers to implementation of paramedic-performed prehospital ultrasound include costs of training, lack of consensus of a training regimen, and complexity involved in scaling up training to large prehospital systems.^20,21^

One solution to address these obstacles and increase access to prehospital ultrasound is to harness real-time data transmission technology,^22^ which would enable a paramedic to communicate with a remote provider with ultrasound experience. Utilizing off-site experts can be useful in environments that are resource-constrained or restricted due to strict isolation precautions. For instance, tele-guided ultrasound has been used by non-physician astronauts on the International Space Station.^23,24^ The concept of tele-ultrasound in the prehospital setting was initially devised in 2008 by Robosoft Inc. (Udupi, India) who developed a portable robot remotely controlled by physicians in France to conduct paramedic-assisted prehospital ultrasound examinations on remote patients in the Mediterranean Sea.^25^ Since then, tele-ultrasound has been evaluated for the assessment or diagnosis of numerous clinical indications, including fetal structural abnormalities,^26–28^ cardiac dysfunction,^29–31^ acute trauma,^32,33^ coronavirus disease 2019/COVID-19,^34^ hepatic and biliary diseases,^35^ thyroid nodules,^36^ breast abnormalities,^37^ dermatologic lesions,^38^ and spinal alterations.^39^ In addition, tele-ultrasound has primarily been studied in low-income rural communities^35,36^ and resource-constrained settings,^30,39–44^ but also in the intensive care unit (ICU)^45,46^ and emergency department (ED).^31,47,48^ Acceptable standards of ultrasound can be taught successfully via tele-guidance to ICU nurses and other non-physician personnel, including ultrasound-naïve firefighters and even non-medical undergraduate students.^49–51^

Because of the increasing interest in tele-ultrasound coupled with a limited understanding of the current evidence on prehospital tele-ultrasound involving paramedics, the authors sought to conduct a scoping review to provide an overview of the literature. This review qualitatively analyzes literature from January 1, 2010 – December 31, 2021 in the MEDLINE (US National Library of Medicine, National Institutes of Health; Bethesda, Maryland USA), EMBASE (Elsevier; Amsterdam, Netherlands), and Cochrane (Wiley; Hoboken, New Jersey USA) online databases on prehospital, paramedic-acquired tele-ultrasound. In addition to assessing image acquisition, image quality, training of tele-ultrasound, and the quality of scientific evidence available, the goals of this review are to summarize current evidence and evaluate the feasibility of paramedic-performed tele-ultrasound in the prehospital setting. The review is geared toward prehospital personnel considering the benefits and costs of implementing tele-ultrasound in their practice and standards of care.

## Methods

**Figure 1.**
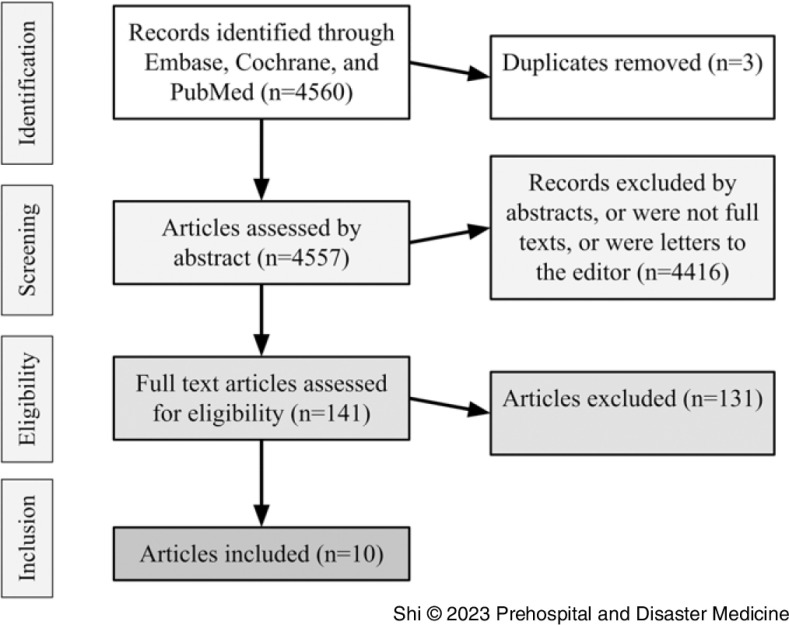
PRISMA Flow Diagram of Article Selection Process.

A systematic article search (Figure 1) was conducted in the MEDLINE, EMBASE, and Cochrane databases for articles during the period from January 1, 2010 – December 31, 2021 using search terms with variations of “ultrasound,” “tele-ultrasound,” “paramedic,” “emergency,” “sonography,” and “prehospital.” The complete search string is provided in the Supplementary Material (available online only). Two reviewers screened articles for inclusion. Included articles pertained to paramedic-performed ultrasound in the prehospital setting and had a tele-medicine component (ie, some form of real-time communication between the paramedic and a remote provider during the ultrasound examination). Retrospective, prospective, and randomized trials and review articles were included because of the lack of randomized controlled trials in the field. All patient ages were included as were all medical and trauma patients. Articles published outside of the range or those with no paramedic on the team were excluded. Case reports/series, abstracts only, editorials, and letters to the editor were excluded. Subsequently, a qualitative synthesis of data was performed, examining study methodology, image acquisition, image quality, and amount of training.

## Results

There were 10 articles (Table 1) that met inclusion criteria. Except for one qualitative survey, the articles could be classified into three major groupings (Table 2): (1) tele-ultrasound involving a specialized tele-echography robot; (2) tele-guidance of an ultrasound-naïve examiner; and (3) remote interpretation of ultrasound images acquired independently by a paramedic. The tele-echography robot system enabled paramedics to quickly attach the robot to the patient so that a FAST exam could be completed by a physician remotely operating the ultrasound probe.^52,53^ While the robot-assisted scans would free the paramedic to provide other forms of medical care, it was the most resource-intensive method and difficult to replicate.

**Table 1.** Prehospital Paramedic-Performed Tele-Ultrasound Study Features

Article	Study Design and Setting	Objective	Number and Experience Level of Paramedic Sonographers	Number of Patients and Patient Population	US Views Obtained	Outcome Measures	Results
Boniface, et al 2011	Feasibility study, simulated prehospital setting, USA	Assess ability of novice paramedics to perform FAST exam under remote guidance	51 US-naive paramedics (20-minute US lecture)	1 healthy volunteer	FAST exam for hemoperitoneum only	Adequacy of images, time to complete examination	100% success rate of obtaining adequate views and FAST images obtained in less than five minutes
Ito, et al 2011	Feasibility study, simulated prehospital setting, Japan	Evaluate performance of a wearable tele-echography robot within an emergency vehicle	9 US-naive paramedics	3 healthy volunteers	FAST exam ^◊^	Time to attach robot, image quality	Robot could be attached by paramedic in less than five minutes, images were of sufficient quality
Ito, et al 2012	Feasibility study, simulated prehospital setting, Japan	Evaluate performance of a wearable tele-echography robot within an emergency vehicle	9 US-naive paramedics	3 healthy volunteers	FAST exam ^◊^	Time to attach robot, time to complete FAST exam	Robot could be attached by paramedic in less than five minutes, FAST scan completed by remote physician in nine minutes, suitable for FAST and emergency medical care
McBeth, et al 2013	Feasibility study, Canada	Evaluate ability of US-naive examiners to identify lung sliding and intraperitoneal fluid with remote mentoring	5 US-naive EMTs	1 healthy volunteer and a phantom model	Four-quadrant FAST exam and extended FAST view of anterior chest	Image quality (five-point Likert score), agreement between expert reviewers	Experts deemed images obtained by EMTs to be adequate (Likert score above four in all views) and clinically useful; participates viewed tele-mentoring positively
Song, et al 2013	Feasibility study, simulated prehospital setting, Korea	Determine whether images obtained by EMTs with ALS training and transmitted from ambulance could be helpful for the diagnosis of hemoperitoneum	1 EMT with ALS and FAST experience	2 anatomical phantoms	FAST exam ^◊^	Sensitivity, specificity, and accuracy of detecting abnormal US results	High sensitivity (90%) and specificity (85.3%), remote tele-ultrasound feasible
Becker, et al 2017	Feasibility study, two urban tertiary ambulance stations, USA	Determine the feasibility of paramedic performed prehospital lung US with remote interpretation in patients with respiratory distress	17 paramedics with two hours of US training	34 patients	Bilateral anterior and lateral views of chest	Agreement between EMS physicians, US experts, and ED diagnosis; interpretability/ transmission success rate of scans	Agreement was only fair; image transmission successful in 73.5% of cases, and 58.8% scans were deemed uninterpretable; tele-ultrasound not feasible based on rigorous criteria
Marsh- Feiley, et al 2018	Qualitative study, interviews, UK	Survey paramedic and physician perspectives on remotely-supported prehospital ultrasound	4 paramedics with one session of US practice			Views on US in context of emergency medicine, legitimacy of remote support in prehospital setting, and anticipated pitfalls and solutions	Paramedics felt optimistic and enthusiastic about prehospital tele-ultrasound while physicians were more skeptical; success depends on effective communication and trust, level of skill, and contextual issues
Morchel, et al 2018	Feasibility study, academic medical center, USA	Assess usefulness of extended FAST images obtained by EMTs	EMTs with 12 hours of US training	20 patients	FAST exam ^◊^	Image quality by 11 Questionnaire for User Interaction Satisfaction Scales	Slightly higher ratings of hospital images but statistically insignificant; ambulatory images essentially as good as quality of hospital bedside images
Nadim, et al 2021	Descriptive feasibility study, university hospital, Denmark	Determine feasibility of “treat and release” strategy for patients with respiratory insufficiency	100 EMTs/ paramedics with six hours of US training	81 patients	Anterior and lateral zones of each hemithorax	On-scene time, feasibility	Feasible for EMTs/paramedics to perform prehospital ultrasound and blood analyses on patients with COPD and release patients following treatment; ambulances spent longer time on scene (70 minutes) to complete the intervention
Leviter, et al 2022	Feasibility study, tertiary children’s hospital, USA	Evaluate effectiveness of a curriculum involving teleguidance in training paramedics to perform US to confirm endotracheal tube placement	4 US-naive paramedics	No information provided	Tracheal and bilateral thoracic views	Pre-test and post-test scores, ability to master lung sliding and tracheal US evaluation, learner feedback on remote curriculum	Paramedic test scores increased when completing the curriculum and were sustained for three months; a virtual curriculum with US tele-guidance is an effective tool to train paramedics in confirmation of endotracheal tube placement

Abbreviations: FAST, Focused Assessment with Sonography for Trauma; US, ultrasound; EMT, emergency medical technician; ALS, Advanced Life Support; EMS, Emergency Medical Services; ED, emergency department; COPD, chronic obstructive pulmonary disease.

**Table 2.** Comparing Three Approaches to Paramedic Prehospital Tele-Ultrasound

Category	Pros	Cons
A paramedic attaches a special tele-echography robot that is remotely controlled by a physician who conducts the exam	• Frees paramedic to provide additional medical care while physician performs scan • Portable robotic system for tele-ultrasound, compatible with body motions	• Difficult to replicate in resource-limited settings • Complexity and cost of equipment • Time-consuming • Not as practical for patients with larger body sizes • No data on usefulness of method for female patients • Remote robot operator not always available immediately
A paramedic performs the scan and is “tele-mentored” in real-time by an ultrasound expert in another location	• Less training required • Lower cost, uses increasingly more accessible technology (laptop, camera, internet) • Potentially more useful in rural and resource-limited settings	• Time-intensive, relatively unsafe • Remote image receiver and mentor not always available immediately • Requires strong verbal communication between medic and remote expert
A paramedic performs the scan which is transmitted in real-time to a receiving ultrasound expert for immediate interpretation	• Safest option, might take less time • Less technological equipment required, more space available in the ambulance	• Moderate training required • Remote image receiver and interpreter not always available immediately

The tele-guidance system involved ultrasound-naïve paramedics performing FAST examinations under the remote guidance of experienced emergency physicians.^54–56^ Tele-guidance would entail less training for the paramedics, but would require strong communication between the “mentor” and paramedic. In particular, a good understanding of anatomic relationships and a “common language” for fanning the probe, switching locations, and probe adjustments in the different views would be key to more successful tele-guidance. In addition, the tele-mentoring studies all utilized healthy volunteers in a stationary setting with reliable network connection, so there was not the added stressor of providing time-sensitive care in a moving ambulance, which could affect communication and internet signal.

Finally, in the approach using remote interpretation, paramedics independently performed the ultrasound scan while images or video were transmitted in real time to a remote physician or ultrasound expert who would interpret the findings.^20,57–59^ Remote image interpretation appeared to be the safest method, but required physicians who were available to interpret, which was not always possible in busy EDs. This complication could be addressed by asking paramedics to limit discussion to a brief pre-alert if the ED is busy.

### Image Acquisition

All studies reported that images could be obtained successfully by paramedics. The majority of images could also be transmitted successfully to remote physicians (the lowest value being 73.5%).^20^ The vast majority found that images were clinically useful or could aid in the diagnosis of the disease of interest. Of the studies that measured the amount of time to scan, the average time spent for paramedics to scan was less than five minutes, which was deemed an adequate amount of time in the prehospital setting.^54^

### Technology

There was tremendous heterogeneity in technological equipment used across the studies (Table 3). For instance, methods of transferring the ultrasound image data included consumer-level smartphones,^20,55–57^ Live-U (LiveU Inc.; Hackensack, New Jersey USA) device,^58^ MPEG-2 compression technology,^52,53^ Skype (Skype Technologies; Luxembourg City, Luxembourg) streaming,^55^ GrandTec (GrandTec USA; Dallas, Texas USA) frame grabber,^57^ or the Lumify app (Philips; Amsterdam, The Netherlands) and React-Secure-app (Meta Platforms, Inc.; Menlo Park, California USA).^59^

**Table 3.** Additional Prehospital Tele-Ultrasound Study Features

Article	US Machine	Recording and Transmission Technology	Capture, Resolution, and Data Transfer Rate
Boniface, et al 2011	SonoSite MicroMaxx, M-Turbo (Fujifilm)	Physician communicating with paramedic via two-way radio and viewing cable video feed from US machine on a monitor	
Ito, et al 2011	SonoSite MicroMaxx (Fujifilm) on a Unique “FASTele” Robot	Video camera, laptop, and WiMAX mobile broadband network for transmission; MPEG-2 compression technology	70 Mbps max
Ito, et al 2012	SonoSite MicroMaxx (Fujifilm) on a Unique “FASTele” Robot	Video camera, laptop, and WiMAX mobile broadband network for transmission; MPEG-2 compression technology	10 Mb/s upload, 40Mb/s download; 25-30fps frame rate
McBeth, et al 2013	SonoSite NanoMaxx (Fujifilm)	Headset for two-way voice communication, video camera mounted on EMT’s head, 3G T-Mobile rocket stick for internet; Skype streaming to transmit video images to receiving Apple iPhone 4 or laptop computer for remote viewing of US images	1.3-megapixel Web camera
Song, et al 2013	SonoSite Titan (Fujifilm)	Notebook computer connected to 3G network through an Apple iPhone 4; GrandTec frame grabber converted US images to digital images for computer, transmitted in JPEG image file format, reconstructed to AVI video file format	640x480 pixel resolution digital images
Becker, et al 2017	SonoSite iViz (Fujifilm)	T-Mobile hotspot device transmitted US images to online archiving system (Trice Medical Imaging Company) which could be accessed by remote physicians	
Morchel, et al 2018	SonoSite M-Turbo (Fujifilm)	Live-U device for video compression and multiplexing over multiple cellular networks for enhanced reliability	
Nadim, et al 2021	Lumify C5-2 (Philips)	Samsung Galaxy G960 smartphone with Lumify-app and React-Secure app platform for secure instant file transfer; Apple iPhone 8 with React-Secure app for video supervision and conferencing	
Leviter, et al 2022	Butterfly IQ+		

Abbreviations: US, ultrasound; EMT, emergency medical technician.

Studies involving tele-guidance through verbal communication utilized two-way radio,^54^ Skype, or the React-Secure-app, which had interactive video conferencing capability. In this way, some of the ultrasound mentors could view the paramedic’s probe position in addition to the ultrasound image on the screen while others could only see the scan. Communication was affected by the amount of background noise, and scanning efficiency was improved when the paramedic had a good understanding of anatomic relationships and when there was a “common language” for a pre-determined starting point, fanning the probe, and switching locations.

### Image Quality

The majority of studies found that paramedics minimally trained in ultrasound could obtain images with adequate quality for interpretation.^54,55,57–59^ Morchel, et al specifically compared the quality of images performed by emergency medical technicians (EMTs) minimally trained in FAST ultrasound to images obtained by in-hospital physicians, and the EMT-acquired images were rated essentially as good as the hospital images. Only one study noted the majority of images (58.8%) obtained by paramedics to be uninterpretable.^20^

### Amount of Training

There were differences in the amount of ultrasound training that paramedics received based on the tele-ultrasound approach. Robot-assisted tele-ultrasound and tele-mentoring studies involved paramedics with minimal training (20 minutes or less), while paramedics who independently scanned had at least two hours of ultrasound training (range: 2-12 hours of training). An average could not be calculated because some studies did not report the number of paramedics who performed scans or the specific number of hours of ultrasound training the EMTs received.^57,58^

One study found that tele-ultrasound with remote guidance was a helpful activity in prehospital ultrasound training for paramedics, which would be applicable for training in any resource-constrained environment without access to on-site ultrasound instructors.^56^

### Paramedic Perspectives on Tele-Ultrasound

One qualitative study explored the perspectives of a small sample of eight paramedics on tele-ultrasound.^60^ The paramedics were optimistic about the technology and saw tele-ultrasound as logical progression from standard POCUS, given advancements in telemetry of other diagnostic tests, such as electrocardiogram/ECG telemetry. On the other hand, physicians were concerned about cost-effectiveness, skill atrophy in rural settings, and usefulness in urban environments with short transport times. Overall, there was a call toward bridging “research enthusiasts and clinical pragmatists” as there is a clear research-practice gap in opinions on tele-ultrasound.

### Feasibility

A list of criteria for determining feasibility of tele-ultrasound was devised by Becker, et al and is shown below:

- Paramedics must successfully obtain images in >80% of attempted cases;
- Expert sonographers must deem images interpretable in >80% of cases;
- Real-time image transmission must be successful in >80% of scans;
- Scans will be clinically useful (ie, ultrasound images correlate with ED diagnosis in >80% of patients).

Only three of the ten studies involved patients in the prehospital setting and could have the criteria applied. One of those studies used tele-ultrasound solely in the prehospital setting, and the data successfully met the first three threshold criteria, but the patients were not seen in the ED and did not have an ED diagnosis to correlate with the prehospital interpretation.^59^ There were mixed results with data from Becker, et al meeting none of the feasibility criteria and reporting many technical issues that considerably limited patient size. On the other hand, data from Morchel, et al suggested that ambulatory images transmitted in real time were essentially as good as the quality of hospital-acquired images. Overall, there was tremendous heterogeneity in methods and technology used and the amount of ultrasound training for paramedics, which limited additional cross-comparisons.

## Discussion

Tele-ultrasound potentially allows for novice prehospital providers to rapidly triage patients with the assistance of a remotely located ultrasound expert and make prompt decisions on patient transport to appropriate facilities. Tele-ultrasound performed by paramedics shows potential to improve ultrasound accessibility and care in the prehospital setting. Research on prehospital tele-ultrasound by paramedics is nascent, and additional studies are needed to address technological challenges and determine feasibility as well as benefit to patients. This screening only found ten relevant articles, which may limit the usefulness of the current evidence. Most of the studies had a high degree of bias and were small-scale studies in simulated settings. The between-study heterogeneity and the lack of control groups and randomized controlled trials hindered cross-study comparisons and meta-analyses. Overall, there was considerable heterogeneity of clinical models, communication methods, and amount of ultrasound experience in the paramedics. The current lack of sufficient and quality evidence on paramedic prehospital tele-ultrasound indicates a pressing need for additional investigation to provide clarity on its feasibility.

Barriers to real-world implementation are numerous and include cost of equipment, difficulties in training, the absence of a remote image receiver and interpreter at the time of examination, uninterpretable images, possibility of equipment failure, patient refusal, and patient acuity.^20^ Common concerns about tele-ultrasound with their respective potential solutions are shown in Table 4. To work around the issue of equipment complexity specifically, some studies are adapting existing broadcast technology for medical diagnostics and rescue. For instance, the same Live-U unit for digital video stream in one tele-ultrasonography study is used in over 60 countries to cover major news and sports events.^61^ In addition, commercial transmission equipment (eg, Live-U) may be better optimized to prevent system overloading (associated with mass-casualty events) and signal dropouts compared to consumer-level smartphones and other non-robust transmission systems.

**Table 4.** Concerns about Prehospital Tele-Ultrasound

Concern	Possible Solutions
Risk to Patient Safety	Decrease scan time while optimizing scanning and interpretation accuracy
Insufficient Time	Common language for verbal communication of fanning probe and switching location; strong understanding of anatomical relationships
Complexity/Cost of Equipment	Adapt existing technology for medical diagnostics (eg, Live-U digital video stream^1^)
Absence of Remote Image Receiver or Interpreter at the Time of Examination	Train emergency department physicians to have more interpreters, minimize hospital crowding; if no interpreters available, ask paramedics to limit discussion to brief pre-alert
Uninterpretable Images or Equipment Failure^2^	Use multiple broadband networks to prevent connection loss; use technology that controls image bit rate depending on network speed

The real-time image transmission rate and time to complete the FAST scan in the studies analyzed in this review appear to be consistent with that of other tele-ultrasound studies.^61^ Other studies have reported no difference in image quality between images transmitted under cellular versus satellite networks.^61^ The studies that reported duration of the FAST scan found that paramedics could complete scans on average under five minutes, which is similar to the time to complete an examination in ED.^62^ It is important to note though that the paramedics scanned under simulated, idealized conditions with healthy volunteers.

**Figure 2.**
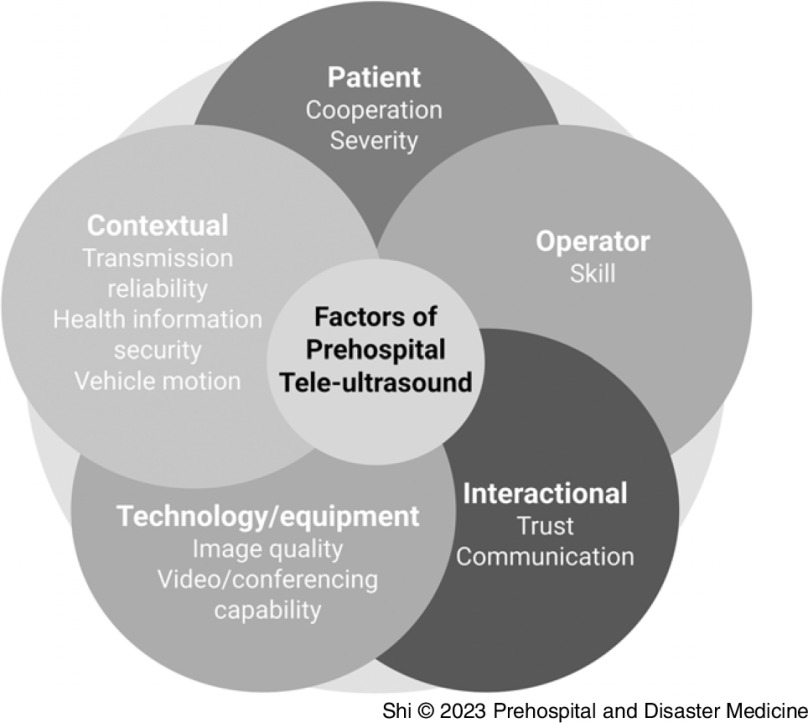
The 5 Pillars of Prehospital Tele-Ultrasound.

The implementation of prehospital tele-ultrasound in different organizations/standards of care depends on numerous factors related to the patient, ultrasound operator, interactions between the operator and remote mentor, technology available, and environment (Figure 2). One framework of understanding the complex integration of novel health care interventions, especially within telehealth and multidisciplinary fields, is normalization process theory. The theory considers different aspects of the technology: coherence (differentiating the technology from existing practices), internalization (seeing benefit or value in the technology), communal and individual specification (how individuals make sense of the technology), cognitive participation (the training and implementation), and collective action (contextual and relational integration, effects on workflow, use of resources).^60^ The likelihood of success in implementing prehospital tele-ultrasound is influenced by these interconnected factors.

## Limitations

This study is a descriptive analysis without a formal bias assessment, and meta-analysis could not be conducted due to study heterogeneity. English-only literature focus and publication bias in the screening could have failed to capture international or unpublished studies.

## Conclusion

Portable tele-ultrasonography could be a solution to save time by providing immediate real-time ultrasound that reduces time-to-diagnosis. With potential applications in resource-limited settings, global health, disaster situations, and acute trauma, where reducing time to definite care is of the essence, prehospital tele-ultrasound may not only reduce time to diagnosis, but also help with accurate patient treatment or referral. Research on prehospital tele-ultrasound by paramedics is nascent, and additional studies are needed to address technological challenges and determine feasibility, benefit to patients, and long-term skill retention.

## Supplementary material

For supplementary material accompanying this paper visit https://doi.org/10.1017/S1049023X23006234. click here to view supplementary material

## References

[1] Rudolph SS , Sørensen MK , Svane C , Hesselfeldt R , Steinmetz J. Effect of prehospital ultrasound on clinical outcomes of non-trauma patients—a systematic review. Resuscitation. 2014;85(1):21–30. 24056394 10.1016/j.resuscitation.2013.09.012

[2] Press GM , Miller SK , Hassan IA , et al. Prospective evaluation of prehospital trauma ultrasound during aeromedical transport. J Emerg Med. 2014;47(6):638–645. 25281177 10.1016/j.jemermed.2014.07.056

[3] Quick JA , Uhlich RM , Ahmad S , Barnes SL , Coughenour JP. In-flight ultrasound identification of pneumothorax. Emerg Radiol. 2016;23(1):3–7. 26407979 10.1007/s10140-015-1348-z

[4] Lichtenstein DA. Blue-protocol and falls-protocol: two applications of lung ultrasound in the critically ill. Chest. 2015;147(6):1659–1670. 26033127 10.1378/chest.14-1313

[5] Netherton S , Milenkovic V , Taylor M , Davis PJ. Diagnostic accuracy of eFAST in the trauma patient: a systematic review and meta-analysis. CJEM. 2019;21(6):727–738. 31317856 10.1017/cem.2019.381

[6] O’Dochartaigh D , Douma M , Alexiu C , Ryan S , MacKenzie M. Utilization criteria for prehospital ultrasound in a Canadian critical care helicopter emergency medical service: determining who might benefit. Prehosp Disaster Med. 2017;32(5):536–540. 28464969 10.1017/S1049023X1700646X

[7] Egan G , Healy D , Neill H , Clarke-Moloney M , Grace PA , Walsh SR. Ultrasound guidance for difficult peripheral venous access: systematic review and meta-analysis. Emerg Med J. 2013;30(7):521. 22886890 10.1136/emermed-2012-201652

[8] Oliveira L , Lawrence M. Ultrasound-guided peripheral intravenous access program for emergency physicians, nurses, and corpsmen (technicians) at a military hospital. Mil Med. 2016;181(3):272–276. 26926753 10.7205/MILMED-D-15-00056

[9] Weston M , Elmer D , McIntosh S , Lundgreen Mason N. Using formalin embalmed cadavers to teach fracture identification with ultrasound. BMC Med Educ. 2020;20(1):227. 32682422 10.1186/s12909-020-02148-8 PMC7368716

[10] Waterbrook AL , Adhikari S , Stolz U , Adrion C. The accuracy of point-of-care ultrasound to diagnose long bone fractures in the ED. Am J Emerg Med. 2013;31(9):1352–1356. 23891601 10.1016/j.ajem.2013.06.006

[11] Lema PC , O’Brien M , Wilson J , et al. Avoid the goose! Paramedic identification of esophageal intubation by ultrasound. Prehosp Disaster Med. 2018;33(4):406–410. 30129915 10.1017/S1049023X18000651

[12] Hanlin ER , Zelenak J , Barakat M , Anderson KL. Airway ultrasound for the confirmation of endotracheal tube placement in cadavers by military flight medic trainees – a pilot study. Am J Emerg Med. 2018;36(9):1711–1714. 29478724 10.1016/j.ajem.2018.01.074

[13] Aletreby W , Alharthy A , Brindley PG , et al. Optic nerve sheath diameter ultrasound for raised intracranial pressure: a literature review and meta-analysis of its diagnostic accuracy. J Ultrasound Med. 2021;41(3):585–595. 33893746 10.1002/jum.15732

[14] Houzé-Cerfon C-H , Bounes V , Guemon J , Le Gourrierec T , Geeraerts T. Quality and feasibility of sonographic measurement of the optic nerve sheath diameter to estimate the risk of raised intracranial pressure after traumatic brain injury in prehospital setting. Prehosp Emerg Care. 2019;23(2):277–283. 30118380 10.1080/10903127.2018.1501444

[15] Brooke M , Walton J , Scutt D , Connolly J , Jarman B. Acquisition and interpretation of focused diagnostic ultrasound images by ultrasound-naive advanced paramedics: trialing a PHUS education program. Emerg Med J. 2012;29(4):322–326. 21511975 10.1136/emj.2010.106484

[16] Kim CH , Shin SD , Song KJ , Park CB. Diagnostic accuracy of focused assessment with sonography for trauma (fast) examinations performed by emergency medical technicians. Prehosp Emerg Care. 2012;16(3):400–406. 22385014 10.3109/10903127.2012.664242

[17] Walcher F , Kirschning T , Müller MP , et al. Accuracy of prehospital focused abdominal sonography for trauma after a 1-day hands-on training course. Emerg Med J. 2010;27(5):345–349. 20442161 10.1136/emj.2008.059626

[18] Heegaard W , Hildebrandt D , Spear D , Chason K , Nelson B , Ho J. Prehospital ultrasound by paramedics: results of field trial. Acad Emerg Med. 2010;17(6):624–630. 20491683 10.1111/j.1553-2712.2010.00755.x

[19] Bøtker MT , Jacobsen L , Rudolph SS , Knudsen L. The role of point of care ultrasound in prehospital critical care: a systematic review. Scand J Trauma Resusc Emerg Med. 2018;26(1):51. 29940990 10.1186/s13049-018-0518-x PMC6019293

[20] Becker TK , Martin-Gill C , Callaway CW , Guyette FX , Schott C. Feasibility of paramedic performed prehospital lung ultrasound in medical patients with respiratory distress. Prehosp Emerg Care. 2018;22(2):175–179. 28910212 10.1080/10903127.2017.1358783

[21] Taylor J , McLaughlin K , McRae A , Lang E , Anton A. Use of prehospital ultrasound in North America: a survey of emergency medical services medical directors. BMC Emerg Med. 2014;14(1):1–5. 24580744 10.1186/1471-227X-14-6 PMC3941255

[22] Adams SJ , Burbridge B , Obaid H , Stoneham G , Babyn P , Mendez I. Telerobotic sonography for remote diagnostic imaging: narrative review of current developments and clinical applications. J Ultrasound Med. 2021;40(7):1287–1306. 33058242 10.1002/jum.15525

[23] Kwon D , Bouffard JA , van Holsbeeck M , et al. Battling fire and ice: remote guidance ultrasound to diagnose injury on the international space station and the ice rink. Am J Surg. 2007;193(3):417–420. 17320547 10.1016/j.amjsurg.2006.11.009

[24] Hamilton DR , Sargsyan AE , Martin DS , et al. On-orbit prospective echocardiography on international space station crew. Echocardiography. 2011;28(5):491–501. 21535119 10.1111/j.1540-8175.2011.01385.x

[25] Fonte A , Essomba T , Vieyres P , et al. *Robotic Platform for an Interactive Tele-echo-graphic System: the PROSIT ANR-2008 Project*. Paper presented at: Hamlyn Symposium on Medical Robotics; May 2010.

[26] Rabie NZ , Sandlin AT , Barber KA , et al. Teleultrasound: how accurate are we? J Ultrasound Med. 2017;36(11):2329–2335. 28660654 10.1002/jum.14304

[27] Rabie NZ , Sandlin AT , Ounpraseuth S , et al. Teleultrasound for pre-natal diagnosis: a validation study. Australas J Ultrasound Med. 2019;22(4):248–252. 34760566 10.1002/ajum.12175 PMC8411791

[28] Whittington JR , Hughes DS , Rabie NZ , et al. Detection of fetal anomalies by remotely directed and interpreted ultrasound (teleultrasound): a randomized noninferiority trial. Am J Perinatol. 2022;39(2):113–119. 34808687 10.1055/s-0041-1739352

[29] Salerno A , Kuhn D , El Sibai R , Levine AR , McCurdy MT. Real-time remote tele-mentored echocardiography: a systematic review. Medicina (Kaunas). 2020;56(12):668. 33276628 10.3390/medicina56120668 PMC7761589

[30] Kaneko T , Kagiyama N , Nakamura Y , et al. Effectiveness of real-time tele-ultrasound for echocardiography in resource-limited medical teams. J Echocardiogr. 2022;20(1):16–23. 34347261 10.1007/s12574-021-00542-9 PMC8335714

[31] Jensen SH , Weile J , Aagaard R , et al. Remote real-time supervision via tele-ultrasound in focused cardiac ultrasound: a single-blinded cluster randomized controlled trial. Acta Anaesthesiol Scand. 2019;63(3):403–409. 30328094 10.1111/aas.13276

[32] Eder PA , Reime B , Wurmb T , Kippnich U , Shammas L , Rashid A. Prehospital telemedical emergency management of severely injured trauma patients. Methods Inf Med. 2018;57(05/06):231–242. 30875702 10.1055/s-0039-1681089

[33] Al-Kadi A , Dyer D , Ball CG , et al. User’s perceptions of remote trauma tele-sonography. J Telemed Telecare. 2009;15(5):251–254. 19590031 10.1258/jtt.2009.081007

[34] Wu S , Wu D , Ye R , et al. Pilot study of robot-assisted teleultrasound based on 5G network: a new feasible strategy for early imaging assessment during COVID-19 pandemic. IEEE Trans Ultrason Ferroelectr Freq Control. 2020;67(11):2241–2248. 32881685 10.1109/TUFFC.2020.3020721 PMC8544926

[35] Marini TJ , Oppenheimer DC , Baran TM , et al. Testing tele-diagnostic right upper quadrant abdominal ultrasound in Peru: a new horizon in expanding access to imaging in rural and underserved areas. PLoS One. 2021;16(8):e0255919. 34379679 10.1371/journal.pone.0255919 PMC8357175

[36] Marini TJ , Weiss SL , Gupta A , et al. Testing tele-diagnostic thyroid ultrasound in Peru: a new horizon in expanding access to imaging in rural and underserved areas. J Endocrinol Invest. 2021;44(12):2699–2708. 33970434 10.1007/s40618-021-01584-7 PMC8572222

[37] Sun YK , Li XL , Wang Q , et al. Improving the quality of breast ultrasound examination performed by inexperienced ultrasound doctors with synchronous tele-ultrasound: a prospective, parallel controlled trial. Ultrasonography. 2021;41(2):307–316. 34794212 10.14366/usg.21081 PMC8942725

[38] Alfageme F , Minguela E , Martínez C , et al. Dermatologic ultrasound in primary care: a new modality of tele-dermatology: a prospective multicenter validation study. J Ultrasound Med. 2021;40(2):351–356. 32767579 10.1002/jum.15409

[39] Marshburn TH , Hadfield CA , Sargsyan AE , Garcia K , Ebert D , Dulchavsky SA. New heights in ultrasound: first report of spinal ultrasound from the international space station. J Emerg Med. 2014;46(1):61–70. 24135505 10.1016/j.jemermed.2013.08.001

[40] Salerno A , Tupchong K , Verceles AC , McCurdy MT. Point-of-care teleultrasound: a systematic review. Telemed J E Health. 2020;26(11):1314–1321. 32302520 10.1089/tmj.2019.0177

[41] Britton N , Miller MA , Safadi S , Siegel A , Levine AR , McCurdy MT. Tele-ultrasound in resource-limited settings: a systematic review. Front Public Health. 2019;7:244. 31552212 10.3389/fpubh.2019.00244 PMC6738135

[42] Robertson TE , Levine AR , Verceles AC , et al. Remote tele-mentored ultrasound for non-physician learners using facetime: a feasibility study in a low-income country. J Crit Care. 2017;40:145–148. 28402924 10.1016/j.jcrc.2017.03.028

[43] Otto C , Shemenski R , Scott JM , Hartshorn J , Bishop S , Viegas S. Evaluation of tele-ultrasound as a tool in remote diagnosis and clinical management at the Amundsen-Scott south pole station and the McMurdo research station. Telemed J E Health. 2013;19(3):186–191. 23480714 10.1089/tmj.2012.0111

[44] Pian L , Gillman LM , McBeth PB , et al. Potential use of remote tele-sonography as a transformational technology in under-resourced and/or remote settings. Emerg Med Int. 2013;2013:986160. 23431455 10.1155/2013/986160 PMC3568862

[45] Duan S , Liu L , Chen Y , et al. A 5G-powered robot-assisted tele-ultrasound diagnostic system in an intensive care unit. Crit Care. 2021;25(1):134. 33827638 10.1186/s13054-021-03563-z PMC8025902

[46] Levine AR , McCurdy MT , Zubrow MT , Papali A , Mallemat HA , Verceles AC. Tele-intensivists can instruct non-physicians to acquire high-quality ultrasound images. J Crit Care. 2015;30(5):871–875. 26122274 10.1016/j.jcrc.2015.05.030

[47] Jensen SH , Duvald I , Aagaard R , et al. Remote real-time ultrasound supervision via commercially available and low-cost tele-ultrasound: a mixed methods study of the practical feasibility and users’ acceptability in an emergency department. J Digit Imaging. 2019;32(5):841–848. 30478478 10.1007/s10278-018-0157-9 PMC6737136

[48] Zennaro F , Neri E , Nappi F , et al. Real-time tele-mentored low cost “point-of-care US” in the hands of pediatricians in the emergency department: diagnostic accuracy compared to expert radiologists. PLoS One. 2016;11(10):e0164539. 27749905 10.1371/journal.pone.0164539 PMC5066956

[49] Douglas TM , Levine AR , Olivieri PP , et al. Brief training increases nurses’ comfort using tele-ultrasound: a feasibility study. Intensive Crit Care Nurs. 2019;51:45–49. 30514602 10.1016/j.iccn.2018.11.004

[50] Kirkpatrick AW , McKee I , McKee JL , et al. Remote just-in-time tele-mentored trauma ultrasound: a double-factorial randomized controlled trial examining fluid detection and remote knobology control through an ultrasound graphic user interface display. Am J Surg. 2016;211(5):894–902. 27020901 10.1016/j.amjsurg.2016.01.018

[51] Ramsingh D , Ma M , Le DQ , et al. Feasibility evaluation of commercially available video conferencing devices to technically direct untrained nonmedical personnel to perform a rapid trauma ultrasound examination. Diagnostics (Basel). 2019;9(4):188. 31739422 10.3390/diagnostics9040188 PMC6963664

[52] Ito K , Tsuruta K , Sugano S , Iwata H. *Evaluation of a Wearable Tele-echography Robot System: FASTele in a Vehicle Using a Mobile Network*. Paper presented at: 2011 Annual International Conference of the IEEE Engineering in Medicine and Biology Society; July 2011. 10.1109/IEMBS.2011.6090389 22254750

[53] Ito K , Sugano S , Takeuchi R , Nakamura K , Iwata H. Usability and performance of a wearable tele-echography robot for focused assessment of trauma using sonography. Med Eng Phys. 2013;35(2):165–171. 22613671 10.1016/j.medengphy.2012.04.011

[54] Boniface KS , Shokoohi H , Smith ER , Scantlebury K. Tele-ultrasound and paramedics: real-time remote physician guidance of the focused assessment with sonography for trauma examination. Am J Emerg Med. 2011;29(5):477–481. 20825815 10.1016/j.ajem.2009.12.001

[55] McBeth P , Crawford I , Tiruta C , et al. Help is in your pocket: the potential accuracy of smartphone-and laptop-based remotely guided resuscitative tele-sonography. Telemed E Health. 2013;19(12):924–930. 10.1089/tmj.2013.0034 24138615

[56] Leviter J , Auerbach M , Amick M , et al. Point-of-care ultrasound curriculum for endotracheal tube confirmation for pediatric critical care transport team through remote learning and teleguidance. Air Med J. 2022;41(2):222–227. 35307147 10.1016/j.amj.2021.11.002

[57] Song KJ , Shin SD , Hong KJ , et al. Clinical applicability of real-time, prehospital image transmission for fast (focused assessment with sonography for trauma). J Telemed Telecare. 2013;19(8):450–455. 24197401 10.1177/1357633X13512068

[58] Morchel H , Ogedegbe C , Chaplin W , et al. Evaluation of a novel wireless transmission system for trauma ultrasound examinations from moving ambulances. Mil Med. 2018;183(suppl_1):111–118. 29635573 10.1093/milmed/usx167

[59] Nadim G , Laursen CB , Pietersen PI , et al. Prehospital emergency medical technicians can perform ultrasonography and blood analysis in prehospital evaluation of patients with chronic obstructive pulmonary disease: a feasibility study. BMC Health Serv Res. 2021;21(1):1–12. 33789641 10.1186/s12913-021-06305-7 PMC8011095

[60] Marsh-Feiley G , Eadie L , Wilson P. Paramedic and physician perspectives on the potential use of remotely supported prehospital ultrasound. Rural Remote Health. 2018;18(3):17–35. 10.22605/RRH4574 30207737

[61] Ogedegbe C , Morchel H , Hazelwood V , Chaplin WF , Feldman J. Development and evaluation of a novel, real time mobile tele-sonography system in management of patients with abdominal trauma: study protocol. BMC Emerg Med. 2012;12(1):19. 23249290 10.1186/1471-227X-12-19 PMC3546944

[62] Nelson BP , Chason K. Use of ultrasound by emergency medical services: a review. Int J Emerg Med. 2008;1(4):253–259. 19384639 10.1007/s12245-008-0075-6 PMC2657261

